# Prevalence and overlap of sarcopenia, frailty, cachexia and malnutrition in older medical inpatients

**DOI:** 10.1186/s12877-019-1115-1

**Published:** 2019-04-27

**Authors:** Anne Gingrich, Dorothee Volkert, Eva Kiesswetter, Marta Thomanek, Svenja Bach, Cornel C. Sieber, Yurdagül Zopf

**Affiliations:** 1 Institute for Biomedicine of Aging, Friedrich-Alexander-Universität Erlangen-Nürnberg, Kobergerstraße 60, 90408 Nürnberg, Germany; 2 Department of Medicine 1, Friedrich-Alexander-Universität Erlangen-Nürnberg, Ulmenweg 18, 91054 Erlangen, Germany; 3 Department of Medicine, Kantonsspital Winterthur, Brauerstrasse 15, 8400 Winterthur, Switzerland

**Keywords:** Cachexia, Frailty, Malnutrition, Sarcopenia, Weight loss

## Abstract

**Background:**

Sarcopenia, frailty, cachexia and malnutrition are widespread syndromes in older people, characterized by loss of body tissue and related to poor outcome. The aim of the present cross-sectional study was to assess the prevalence of these syndromes and their overlap in older medical inpatients.

**Methods:**

Patients aged 70 years or older who had been admitted to the internal medical department of a German university hospital were recruited. Sarcopenia, frailty, cachexia and malnutrition were assessed in a standardized manner according to current consensus definitions. Prevalence rates of these syndromes and their constituents and the concurrent occurrence of the syndromes (overlap) were calculated.

**Results:**

One hundred patients (48 female) aged 76.5 ± 4.7 years with a BMI of 27.6 ± 5.5 kg/m^2^ were included. The main diagnoses were gastroenterological (33%) and oncological diseases (31%). Sarcopenia was present in 42%, frailty in 33%, cachexia in 32% and malnutrition in 15% of the patients. 63% had at least one syndrome: 32% one, 11% two, 12% three and 8% all four. All four syndromes are characterized by significant weight loss during the last 12 months, which was most pronounced in malnourished patients and least pronounced in frail patients, and by significantly reduced physical performance. All syndromes were significantly pairwise related, except malnutrition and frailty. In 19% of patients sarcopenia and frailty occurred concurrently, in 20% frailty and cachexia and in 22% sarcopenia and cachexia with or without additional other syndromes. All malnourished patients except one were also cachectic (93%) and 80% of malnourished patients were also sarcopenic. 53% of malnourished patients were in addition frail, and these patients were affected by all four syndromes.

**Conclusions:**

Nearly two thirds of older medical inpatients had at least one of the tissue loss syndromes sarcopenia, frailty, cachexia and malnutrition. The syndromes overlapped partly and were interrelated. Future studies with larger patient groups and longitudinal design are required to clarify the significance of single and concurrent occurrence of these syndromes for clinical outcome and successful therapy.

## Introduction

Sarcopenia, frailty, cachexia and malnutrition are widespread syndromes in older people and associated with an increased risk of poor outcome like mobility limitation, fracture, increased length of hospital stay, hospital readmission, morbidity and mortality [1–4]. In hospital patients the hazard of these syndromes is particularly high due to acute illness, immobility and anorexia. Prevalence rates up to 50% or more have been reported in older patients for all syndromes [5–8], however, with great variation between studies, not least due to of different diagnostic criteria.

One central aspect of all syndromes is the loss of body tissue [9], which however affects different tissues to a varying extent. Whereas in sarcopenia, frailty and cachexia the loss of fat-free mass, especially skeletal muscle mass, is crucial [10–12], in malnutrition fat free mass as well as fat mass are reduced [13]. Besides these changes in body composition, all syndromes except malnutrition are characterized by specific additional features: sarcopenia and frailty by reduced strength and function [10, 11], frailty in addition by exhaustion and low physical activity [11], and cachexia by reduced strength, fatigue, anorexia and inflammation [12]. All syndromes also share similar etiological factors which are differently pronounced, i. e. reduced food intake, inflammation, hormonal changes, increased energy requirements and reduced physical activity [9].

Due to these similarities in etiology and definitions, the syndromes partly overlap and can be present in the same patient. This concurrent occurrence might have implications for adequate treatment but also for outcome. Plenty of studies are available focusing on one specific syndrome, but until now only few studies looked at more than one syndrome in the same population. In geriatric patients, simultaneous occurrence of sarcopenia and malnutrition [5, 14] and of sarcopenia and cachexia [15] was described. In community-dwelling older adults, significant overlaps between sarcopenia and frailty [16–18] and between malnutrition and frailty [19] are reported.

To our knowledge, prevalence and concurrent occurrence of all four aforesaid syndromes in older inpatients have not been investigated before. Thus, the aim of the present study was to assess the prevalence and overlap of sarcopenia, cachexia, frailty and malnutrition in a group of older medical inpatients.

## Methods

### Study design and inclusion criteria

All patients consecutively admitted to the Internal Medical Department 1 of the University Hospital Erlangen in Germany between August 2014 and November 2014 and fulfilling the following inclusion criteria were asked to participate in the present cross-sectional study: age 70 years or older, no severe cognitive impairment, ability to perform Bioelectrical Impedance Analysis (BIA) in a standing position, no end of life situation and ability to communicate and answer questions. The study was approved by the ethics committee of the Friedrich-Alexander-Universität Erlangen-Nürnberg. All participants signed an informed consent form. Data on patient characteristics and components of tissue loss syndromes were collected by two trained medical PhD students (SB, MT) within 72 h of hospital admission.

### Assessment of patients’ characteristics

Age, sex, main medical diagnoses and number of drugs taken were extracted from medical records. Living situation (alone / with other persons / nursing home) was asked in a personal interview. Independence in basic activities of daily living (ADL) was assessed using the Barthel Index with a maximum of 100 points indicating complete independency [20]. Depressive symptoms were screened using the Geriatric Depression Scale (GDS) [21]. The score ranges from 0 to 15, with 15 points indicating severe depressive symptoms. Cognitive status was rated with the Mini-Mental State Examination (MMSE) [22]. A score of 25 or more of 30 possible points indicates normal cognitive status. Comorbidity was assessed using the Charlson Comorbidity Index (CCI) which considers the presence of 19 defined diseases [23]. The highest possible score is 37 points indicating severe comorbidity. Lower extremity function was assessed using the Short Physical Performance Battery (SPPB), comprising tests for balance, gait speed (4 m course) and strength (sit-to-stand, 5 repetitions) [24]. The score ranges from 0 to 12, with 10 or more points indicating high physical performance.

### Definitions of tissue loss syndromes

Sarcopenia, cachexia, frailty and malnutrition were assessed according to established consensus definitions which are widely used.

*Sarcopenia* was defined according to the consensus definition of the European Working Group on Sarcopenia in Older People (EWGSOP) [10] by presence of low muscle mass plus either low handgrip strength or/and poor physical performance defined by slow gait speed.

*Frailty* was assessed according to Fried et al. [11] by presence of three or more of the following five criteria: unintentional weight loss, exhaustion, low physical activity level, slow gait speed and low handgrip strength.

*Cachexia* was defined according to Evans et al. [12] by weight loss in the presence of illness, combined with three or more of the following five criteria: decreased handgrip strength, fatigue, anorexia, low FFMI or abnormal biochemistry (high CRP, low Hb or low albumin). For two participants body weight 12 months ago was not available, instead a BMI < 20.0 kg/m^2^ was considered as recommended by Evans et al. [12].

*Malnutrition* was defined in accordance with the ESPEN Consensus Statement by Cederholm et al. [13]. After screening for malnutrition using the Mini Nutritional Assessment short form (MNA-SF) [25], patients who were at risk of malnutrition or malnourished (12 points) were further rated. Either the presence of a BMI < 18.5 kg/m^2^ was categorized as malnutrition (alternative 1) or the occurrence of unintentional weight loss combined with either a BMI < 22 kg/m^2^ or reduced FFMI (alternative 2).

Diagnostic criteria and the respective cut-off values are summarized in Table 1, assessment of individual components of the syndromes is described below.

**Table 1:** Diagnostic criteria of tissue loss syndromes

	Sarcopenia (EWGSOP [10])	Frailty (Fried et al. [11])	Cachexia (Evans et al. [12])	Malnutrition (ESPEN [13])
Summary	Low SMI + (low grip strength OR slow gait speed)	≥ 3 of 5 criteria	WL + ≥ 3 of 5 criteria	MNA-SF < 12 + [BMI < 18.5 OR [WL + (BMI < 22 OR low FFMI)]]
Weight loss	–	≥10 pounds unintended in previous year	> 5% in previous year	> 10% unintended in previous year
BMI [kg/m^2^]	–	–		< 18.5 / < 22
FFMI [kg/m^2^]	–	–	< 15 (w)/ < 17 (m)	< 15 (w) / < 17 (m)
SMI [kg/m^2^]	≤ 6.75 (w) / ≤10.75 (m)	–		–
Handgrip strength [kg]	≤ 17–21 (w) / ≤ 29–32 (m)^1^	≤17–21 (w) / ≤ 29–32 (m)^1^	≤ 17–21 (w) / ≤ 29–32 (m)^1^	–
Usual gait speed [m/s]	< 0.8	< 0.65 or < 0.76^2^	–	–
Fatigue / exhaustion	–	positive answer to ≥ 1 of 2 questions	FACIT-F < 30 points	–
Physical activity [kcal/week]	–	< 270 (w) / < 383 (m)	–	–
Anorexia	–	–	low intake or poor appetite	–
Biochemistry	–	–	CRP > 5.0 mg/L or Hb < 12 g/dL or albumin < 3.2 g/dL	–

Abbreviations**:** *BW* body weight, *CRP* C-reactive protein, *FACIT-F* Functional Assessment of Chronic Illness Therapy Fatigue Scale [28], *FFMI* fat-free mass index, *Hb* hemoglobin; *m* men, *SM* skeletal muscle index, *WL* weight loss, *w* women

^1^sex- and BMI-specific cut-off value [11]; ^2^ sex- and height-specific cut-off value [11]

### Assessment of components of tissue loss syndromes

Bioelectrical impedance and body weight were measured using a seca medical Body Composition Analyzer 514 (seca, Hamburg, Germany) in a standing position. Body height was measured with a stadiometer, and BMI was calculated. Based on BIA skeletal muscle mass was calculated according to Janssen et al. [26] and divided by height squared to obtain the skeletal muscle index (SMI). Fat-free mass was calculated using the equation by Kyle et al. [27] and divided by height squared to obtain the fat free mass index (FFMI). Patients were asked for their body weight 12 months ago and for their usual body weight. The weight change was then calculated by subtraction from the measured current weight. In the case of weight loss, patients were additionally asked whether this was intentional. For the two participants with missing body weight data from 12 months before, weight change between present and usual body weight was considered for the definition of weight loss in frailty and malnutrition. Anorexia was assessed according to Landi et al. [28] as the presence of decreased food intake and/or the presence of poor appetite during the last days. Fatigue was identified using the Functional Assessment of Chronic Illness Therapy Fatigue Scale (FACIT-F) [29]. The score ranges from 0 to 52 points, with a score of less than 30 indicating severe fatigue. Exhaustion was identified according to Fried et al. [11] by two questions from the Center for Epidemiological Studies–Depression Scale (CESD-S) [30]. Handgrip strength was measured with a JAMAR hydraulic hand dynamometer following the Southampton protocol for adult grip strength measurement [31]. Physical activity was assessed with the Minnesota Leisure Time Activities Questionnaire [32]. Cut-off values for low physical activity were < 383 kcal per week for men and < 270 kcal per week for women.

Albumin, hemoglobin (Hb) and C-reactive protein (CRP) concentrations were analyzed from fasting blood samples within hospital laboratory routine.

### Data analysis and statistics

Results are presented as median and interquartile range or relative frequencies. Data were tested for normality using Kolmogorov-Smirnov test. Due to non-normal distribution, the Mann-Whitney U test was used to test differences in patients’ characteristics between the groups with and without each tissue loss syndrome for statistical significance. The Chi-square test was applied to detect differences between categorical data. In case of a significant result, a Post hoc z-test with Bonferroni correction was applied to locate these differences. Statistical analysis was performed with SPSS Version 23 (IBM SPSS Statistics, Chicago, IL). Level of significance was set at *p* < 0.05.

## Results

### Patients’ characteristics

One hundred patients were included. From 1086 patients admitted, 684 had to be excluded because of age < 70 years, 60 because of inability to perform BIA, 51 because of severe cognitive impairment, 37 because of end of life situation/severe illness and 15 patients were not able to answer questions for other reasons. One hundred thirty nine patients were unwilling to participate.

Median age of the participants was 76.0 years, 48% were female, median BMI was 26.6 kg/m^2^ and 27% were obese (Table 2). The main diagnoses were gastroenterological (33%) and oncological diseases (31%). Thirty-six patients were suffering from other diseases: chronic obstructive pulmonary disease (*n* = 6), other disease of the respiratory tract (n = 6), infection (*n* = 5), disease of the circulatory system (n = 5), disease of the blood (n = 5), endocrine, nutritional or metabolic disease (*n* = 4), diseases of the genitourinary (*n* = 3) and other diseases (*n* = 2). Median CCI was 1 indicating very low comorbidity, nevertheless 59% took 5 or more medications. 93% of patients were completely independent in basic ADL and 49% had a SPPB score of 10 or more points reflecting high physical performance.

**Table 2:** Patients’ characteristics of the total sample and of subgroups of patients with sarcopenia, frailty, cachexia and malnutrition (% or median [IQR])

	All (*n* = 100)	Sarcopenia (*n* = 42)	Frailty (*n* = 33)	Cachexia (*n* = 32)	Malnutrition (*n* = 15)
Sex					
female	48.0	40.5	63.6*	46.7	53.1
Age [years]	76.0 [73.0–79.0]	77.5 [73.0–81.0]	76.0 [73.0–80.5]	76.5 [73.0–79.8]	76.0 [71.0–79.0]
BMI [kg/m^2^]	26.6 [23.8–30.2]	26.7 [22.7–29.7]	28.7 [23.5–32.1]	25.0 [21.1–29.6]*	21.8 [20.5–23.9]*
BMI > 30 kg/m^2^	27.0	21.4	30.3	0.0*	18.8
Living situation					
Alone	23.0	28.6	21.2	26.7	15.6
With others	76.0	71.4	75.8	73.3	84.4
Nursing home	1.0	0.0	3.0	0.0	0.0
Main diagnosis					
Oncological	31.0	33.3	25.0	27.3	43.8
Gastroenterological	33.0	31.0	38.2	30.3	21.9
Other	36.0	35.7	36.8	42.4	34.4
Comorbidity: CCI [points]	1.0 [0.0–2.0]	2.0 [0.0–2.0]	2.0 [0.5–2.5]	2.0 [1.0–3.0]*	2.0 [0.0–2.0]
Number of drugs	6 [3–8]^1^	7.0 [4.0–8.0]^3^	7.0 [5.0–8.5]*	7.0 [4.0–9.0]^2^	5.5 [2.8–7.8]^2^
Polypharmacy (≥ 5 drugs)	59.0^1^	64.3^3^	84.8*	53.3^2^	68.8^2^
Activities of daily living: Barthel index					
Completely independent (100 points)	93.0	90.5	84.8*	93.3	84.4*
Physical performance: SPPB [points]	9.0 [7.0–11.0]	9.0 [6.8–10.3]*	7.0 [4.5–9.0]*	8.5 [4.5–9.8]*	9.0 [7.0–9.0]
Reduced performance (< 10 points)	51.0	66.7*	81.8*	80.0*	75.0*
Cognitive status: MMSE [points]	28.0 [26.0–29.0]^2^	28.0 [26.0–29.0]	28.0 [26.0–29.0]^2^	28.0 [26.0–29.0]^2^	29.0 [28.0–30.0]
Impaired cognition (≤ 24 points)	13.0^2^	14.3	12.1^2^	0	12.5^2^
Depressive symptoms: GDS [points]	2.0 [0.0–3.0]	2.0 [0.0–4.0]	3.0 [1.0–5.5]*	3.0 [1.0–5.0]*	3.0 [1.0–7.0]*
Severe depressive symptoms (≥ 10 points)	2.0	2.4	6.1*	6.7	6.3*

Abbreviations**:** IQR: interquartile range; BMI: body mass index; CCI: Charlson Comorbidty Index (score range 0–37); SPPB: Short Physical Performance Battery (score range: 0–12); MMSE: Mini-Mental State Examination (score range: 0–30); GDS: Geriatric Depression Scale (score range: 0–15); MNA-SF: Mini Nutritional Assessment short form (score range: 0–14); WL, weight loss

^1^4 missing; ^2^ 1 missing; ^3^ 2 missing;

*Significant difference between patients with and patients without the respective syndrome (*p* < 0.05)

### Prevalence rates of tissue loss syndromes and their relation to patient characteristics and components of tissue loss syndromes.

Sarcopenia was present in 42%, frailty in 33%, cachexia in 32% and malnutrition in 15% of the patients. 10% had severe sarcopenia (all 3 criteria) and 36% pre-sarcopenia (low SMI only); 54% were pre-frail (1 or 2 criteria). The prevalence rates of the four syndromes were not significantly different between the three main diagnostic groups (Table 2). Compared to patients without the respective syndrome, physical performance was significantly reduced in all syndromes, and BMI was lower in malnourished and cachectic patients. Patients with frailty and patients with cachexia had more often reduced ADLs than those without these syndromes, and patients suffering from frailty were significantly more often female and took more drugs than patients without frailty. BMI was above 30 kg/m^2^ in nearly one third of frail patients.

Prevalence rates of the components of the four syndromes are presented in Table 3 for the whole group and for the subgroups of patients with each syndrome. All single diagnostic criteria of each syndrome were significantly more prevalent in patients with the respective syndrome than in patients without (e.g. low SMI, low handgrip strength and low gait speed in sarcopenic patients) (indicated in bold in Table 3). In addition, malnourished patients had significantly more often low albumin concentrations than non-malnourished, and frail patients had significantly more often low hemoglobin values and more often anorexia than non-frail patients. In cachectic patients, the BMI was significantly more often reduced than in non-cachectic patients and all except two had a low SMI. All four syndromes are characterized by significant weight loss during the last 12 months, which was most pronounced in malnourished and least pronounced in frail patients. By definition all malnourished and all cachectic patients had weight loss > 5% and all malnourished patients (unintended) weight loss > 10% in the last 12 months. In patients with each syndrome, the prevalence of malnutrition according to MNA was significantly higher compared to those without the respective syndrome.

**Table 3:** Components of tissue loss syndromes in the total sample and in patients with sarcopenia, frailty, cachexia and malnutrition (% or median [IQR])

	All (n = 100)	Sarcopenia (*n* = 42)	Frailty (*n* = 33)	Cachexia (*n* = 32)	Malnutrition (*n* = 15)
WL in last 12 months [kg]	5.1 [0.7–14.1]^1^	10.7 [1.8–16.7]*^1^	9.1 [4.2–16.3]*	14.3 [8.4–17.7*]	17.9 [14.9–21.3]*
WL > 5% in last 12 months	50.0^1^	61.9*^1^	75.8*	100.0*	100.0*
WL > 10% in last 12 months	28.0^1^	47.6*^1^	39.4	56.3*	100.0*
WL > 15% in last 12 months	22.0^1^	35.7*^1^	30.3	43.8*	73.3*
Unintended WL > 10 pounds in last 12 months	36.0^1^	47.6*^1^	63.6*	81.3*	86.7*
Unintended WL > 10% in last 12 months	24.0^1^	40.5*^1^	36.4*	56.3*	100.0*
BMI < 18.5 kg/m2	2.0	4.8	3.0	6.3*	13.3*
BMI < 22 kg/m^2^	14.0	21.4	21.2	31.3*	53.3*
FFMI < 15 (w) / 17 (m) kg/m^2^ [13]	42.0	21.0	42.4	65.6*	100.0*
SMI ≤6.75 (w) / ≤10.75 (m) [49]	78.0	100.0*	69.7	93.8*	100.0*
Low handgrip strength [11]	50.0	85.7*	75.8*	68.8*	66.7
Gait speed < 0.8 m/s [50]	27.0	38.1*	54.5*	34.4	33.3
Gait speed < 0.65 / 0.76 m/s [11]	18.0	21.4	42.4*	28.1	20.0
Exhaustion [11]	46.0	40.5	84.8*	62.5*	53.3
Severe fatigue (FACIT-F < 30 points) [28]	37.0	35.7	69.7*	59.4*	46.7
Low physical activity [31]	45.0	54.8	87.9*	53.1	40.0
Albumin < 3.2 g/dL	18.0	19.0	24.2	37.5*	40.0*
Hemoglobin < 12 g/dL	48.0	52.4	63.6*	**50.0**	60.0
CRP > 5 mg/L	73.0	76.2	72.7	87.5*	86.7
Anorexia [27]	51.0	57.1	66.7*	78.1*	66.7
MNA-SF [points] [32]	11.0 [10.0–12.0]	11.0 [8.0–12.0]	10.0 [7.0–11.5]*	9.0 [7.3–11.0]*	8.0 [6.0–10.0]*
MNA-SF – at risk of malnutrition (8–11 points)	48.0	45.2	48.5	59.4	**53.3**
MNA-SF – malnourished (< 8 points)	11.0	19.0*	27.3*	25.0*	46.7*

Abbreviations: *BMI* body mass index, *CRP* C-reactive protein, *FACIT-F* Functional Assessment of Chronic Illness Therapy Fatigue Scale (score range: 0–52 points), *FFMI*, fat-free mass index, *IQR* interquartile range, *MNA-SF* Mini Nutritional Assessment Short Form (score range: 0–14); SMI, skeletal muscle index; WL, weight loss

^1^2 missing; Bold: diagnostic criteria of the respective syndrome

*Significant difference between patients with and patients without the respective syndrome (p < 0.05)

### Overlap between the tissue loss syndromes

**Fig. 1:**
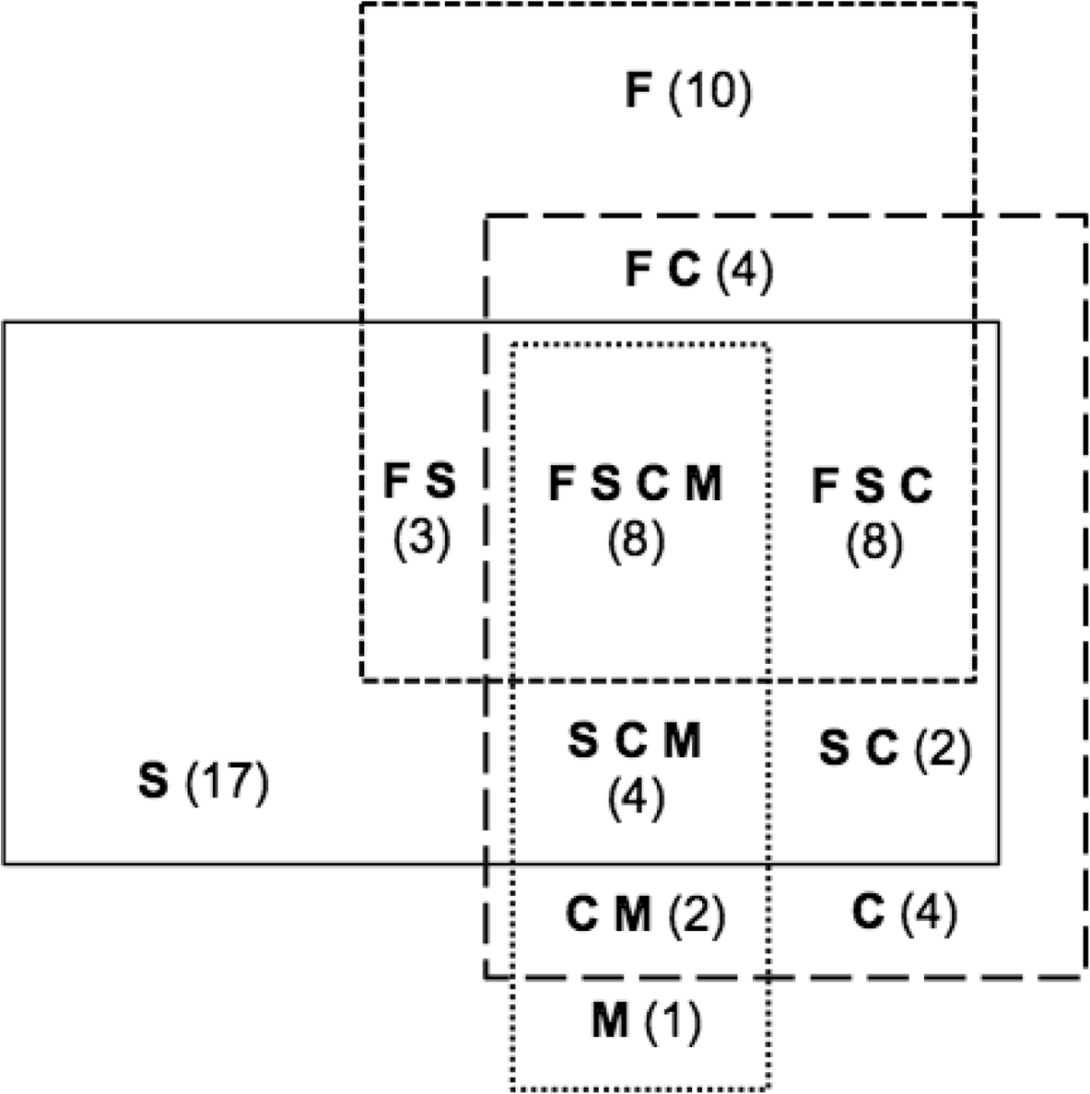
Overlap of sarcopenia , frailty , cachexia , and malnutrition  in older medical inpatients (n=100)

In 63% of patients at least one tissue loss syndrome was observed: in 32% one, in 11% two, in 12% three and in 8% all four. All syndromes were significantly pairwise associated, except malnutrition and frailty (Table 4). The overlap between sarcopenia, cachexia, frailty and malnutrition is illustrated in a Venn diagram in Fig. 1. In 19% of patients, sarcopenia and frailty occurred concurrently, in 20% frailty and cachexia and in 22% sarcopenia and cachexia with or without additional other syndromes. All malnourished patients except one were also cachectic (93%) and 80% of malnourished patients were also sarcopenic. 53% of malnourished patients were also frail, and these patients were affected by all four syndromes.

**Table 4:** Prevalence of other tissue loss syndromes (%) in patients with a specific syndrome

	Sarcopenia (*n* = 42)	Frailty (n = 33)	Cachexia (n = 32)	Malnutrition (n = 15)
Sarcopenia	–	57.6*	68.8*	80.0*
Frailty	45.2*	–	62.5*	53.3
Cachexia	52.4*	60.6*	–	93.3*
Malnutrition	28.6*	24.2	43.8*	–

*Significant difference between patients with and patients without the respective syndrome (p < 0.05)

## Discussion

To the best of our knowledge this is the first study covering the four tissue loss syndromes (sarcopenia, frailty, cachexia and malnutrition) simultaneously in one patient group. Sarcopenia was the most prevalent syndrome in 42% of older medical inpatients and malnutrition the least prevalent in only 15%. Frailty and cachexia were prevalent in about one third respectively. Almost two thirds of the patients were affected by at least one of these syndromes and considerable overlap was observed (Fig. 1). Eight percent had all four syndromes.

The patients were in rather good physical and mental condition, reflected in complete independence in ADLs in almost all patients, only few cognitive impairment, only occasional depressive symptoms and very low comorbidity (Table 2). Consequently, we experienced no difficulties measuring handgrip strength and gait speed which are reported by others in geriatric populations [14]. This positive selection is at least partly due to the inclusion criteria and demands for more comprehensive data assessment in the future.

Prevalence rates of a specific syndrome are generally dependent on the applied definition. In recent years, consensus definitions have been developed for all four syndromes which are widely used in research and were also used in the present study. For the assessment of sarcopenia, the EWGSOP definition [10] was applied because it combines measures of muscle mass and function and its relation to poor clinical outcome is well documented [33]. This definition is well established, and has previously been used in several studies in older hospitalized patients with reported prevalence rates between 10 and 50% [34–38]. A closer look at the methods used in these studies reveals that despite using the same general definition, different operational procedures were used, i.e. different methods to determine muscle mass, and different cut-off values to define reduced muscle mass and function. Using the same diagnostic criteria as in our study, Perez-Zepeda et al. [34] reported sarcopenia in 40% and Sousa et al. [35] in 37% of older patients, which is similar to our results.

Frailty was assessed using the definition of Fried et al. [11], as this definition is most widely used, is focused on the physical phenotype of frailty and relatively straightforward to assess. In our study one third of the patients were found to be frail. This proportion is in the same range as reported earlier by others using the same definition in older patient populations [39–41]. Frailty was observed less often than sarcopenia and only 58% of frail patients were also sarcopenic (Table 4). This phenomenon, i.e. much lower prevalence of frailty than of sarcopenia, is also reported in community-dwelling older adults [16, 18], although not consistently [17]. The frailty definition includes reduced functionality – low grip strength as well as slow gait speed – but is, in contrast to all other syndromes, not necessarily characterized by reduced muscle mass [11] which is confirmed in our results. The most prevalent features of frail patients were exhaustion and low physical activity in 85 and 88% of patients, respectively (Table 3).

Cachexia is widely acknowledged as a complex metabolic syndrome observed in chronic diseases associated with systemic inflammation, but difficult to assess due to the lack of a clear definition for clinical application. We operationalized cachexia according to Evans et al. [12] who included biochemical measures which are regarded as important parameters of the cachexia syndrome and found a prevalence of 32% which is almost exactly the same as reported from palliative care cancer patients (age 68 ± 11 years) using the same definition (33%) [42]. No other studies reporting the prevalence of cachexia in older hospitalized patients were found. This is in accordance with a recent systematic review on the prevalence of cachexia and malnutrition in older cancer patients scheduled for chemotherapy, where 24 studies were identified reporting the prevalence of malnutrition but no study specifically assessed cachexia [43]. Although cachexia is typically attributed to cancer [3], it was not significantly more often observed in patients with a primary diagnosis of cancer (Table 1). Several components of the cachexia definition were also present in patients with other syndromes, e.g. anorexia and low hemoglobin levels in frail patients and reduced body mass, fat-free mass and albumin levels in malnourished patients. Besides the necessary criterion loss of weight, the most prevalent features in cachectic patients were increased CRP values and anorexia in more than three quarters of patients (Table 3).

Malnutrition was assessed according to the ESPEN definition [13], the first consensus definition with age-specific cut off values for a low BMI. Since its publication in 2015, the ESPEN definition was used in a number of studies [5, 44–46] providing a good basis for comparison of our own results. Surprisingly, malnutrition, which is acknowledged as a widespread syndrome in older patients [4], was by far the least prevalent syndrome in only 15% of our patients, less than half as frequent as the other syndromes. This prevalence is, however, in accordance with previous studies using the ESPEN definition, reporting malnutrition in 11 to 15% of somewhat younger hospitalized patients (mean ages between 57 and 62 years) [44–46]. A slightly higher prevalence of 19% was observed in markedly older patients (85 ± 6 years) in a post-acute geriatric rehabilitation care unit [5]. In the above mentioned review on malnutrition and cachexia in older cancer patients, a wide variation in the prevalence of malnutrition was reported when using weight loss (8–40%) as well as MNA (3–42%) as criteria for malnutrition [43]. In our study, the prevalence of malnutrition according to MNA-SF was 11%, thus in the lower part of this range and even lower than found by using the ESPEN definition. This may be explained by the close relation of the MNA to functionality and level of dependence [47] and the low level of dependence in our patient group (Table 1).

Interestingly, malnutrition according to MNA was significantly more prevalent not only in patients with malnutrition according to ESPEN but also in all other syndromes (Table 3). This might also be due to the functional nature of the MNA. Whereas malnutrition according to MNA was related to frailty as reported earlier by others [47], malnutrition according to ESPEN was not (Table 4). This is again explainable by the fact that functional aspects are included in the MNA whereas the ESPEN definition is restricted to weight loss and reduced body mass or fat-free mass. Despite this lacking significance in the association between malnutrition and frailty, malnutrition according to ESPEN showed the largest overlap with the other syndromes (Table 4**,** Fig. 1). All malnourished patients except one were also cachectic, which may be due to the fact that two of the three malnutrition criteria – weight loss and low fat-free mass – are also components of the cachexia definition. Despite completely different diagnostic criteria, however, 80% of the malnourished patients were also sarcopenic. Interestingly, all malnourished patients had a low SMI, probably as a consequence of the experienced weight loss. Thus, the large overlap of malnutrition with the other syndromes is probably caused by the fact that malnutrition is mainly characterized by wasting which is a central symptom also of the other syndromes. Half of all patients reported a weight loss of more than 5% within the last 12 months, and this proportion was significantly higher in all syndromes (Table 3) – also in sarcopenia even though weight loss is not part of the sarcopenia definition.

Interestingly, many more patients in our study had a reduced muscle mass (78%) than reported a weight loss, indicating that muscle mass is often reduced also in persons who did not experience a significant weight loss.

Even though every other patient experienced weight loss, more than one quarter of the patients was obese, and obesity did not preclude the presence of sarcopenia, frailty or cachexia. In these patients, tissue loss is hidden behind high body (fat) mass and may easily be overlooked if no special attention is paid. Obese patients with one or more of the four syndromes might have higher risks for cardio-metabolic diseases and physical disability compared to patients with normal BMI and these syndromes [48] and thus deserve special attention. In order to identify these patients, body composition needs to be measured.

The major strength of the present study is the application of consensus definitions and complete information on all four syndromes in the same patients. Moreover, all measurements were highly standardized. One limitation of the study is the rather small sample size, which is however, comparable to previous studies investigating the overlap of tissue loss syndromes in hospital settings [5, 14]. Due to hospital equipment, we were forced to select patients who were able to stand which limits the generalizability of our results. In addition, 13% of the initially admitted or 58% of eligible patients had to be excluded due to their unwillingness to participate, further restraining generalizability. As these patients did not consent to any data collection, it is unfortunately not possible to describe potential differences between these patients and the study sample. It may be assumed that a selective participation of less severely impaired patients also contributed to the high physical and mental performance of the study sample. Another limitation is that weight loss was assessed based on participant’s self-reported body weight 12 months ago and not measured. In addition, the original definition of weight loss in the ESPEN malnutrition definition (> 10% of habitual weight indefinite of time, or > 5% over 3 months [13]) had to be adapted to > 10% weight loss in the previous year. This might have led to a slight underestimation of the malnutrition prevalence. Furthermore, the accuracy of BIA measurements is limited in case of changes in the amount and distribution of body water.

## Conclusion

In the present cross-sectional study, the tissue loss syndromes sarcopenia, frailty, cachexia and malnutrition and their constituting components were widespread among older medical inpatients, even though patients were physically and mentally rather unimpaired. The syndromes occurred concurrently and were interrelated.

In the light of well-known serious health consequences of each syndrome, in clinical routine attention should be paid to the presence of each syndrome, also in obese patients. In addition, each component of the syndromes needs particular attention as weight loss, reduced muscle mass, reduced physical performance and inflammation are treatable by nutritional support, physical exercise or anti-inflammatory treatment.

Future studies with larger patient groups and longitudinal design are required to clarify the significance of single and concurrent occurrence of these syndromes for clinical outcome and successful therapy. In this context, specific attention should be paid to diagnostic tools and cut-off values, since prevalence as well as overlap of the syndromes is largely dependent on the applied diagnostic criteria.
